# Mr. Chamberlaine, on the Amendments of the Medicine Act

**Published:** 1803-08-01

**Authors:** 


### Mr. Chamberlaine, *on the* Amendments *of the* Medicine Act.

"Non sunt autem pejores laquei, quam laquei legum." Bacon, Resusc.

To Dr. BRADLEY.

Sir,

It is now exactly a twelvemonth ago, since I took the liberty of trespassing on the patience of the Readers of the Medical and Physical Journal, by troubling you with some Observations on the substance and tendency of a new act of parliament, known by the name of the Medicine Act, which, as it then stood, tended greatly to embarrass those gentlemen of the Medical profession who are practitioners in Pharmacy, and more particularly those who from their situation and circumstances, find their account in keeping shops for selling medicines *by retail.*

Very soon after the publication of my letter to you, in

No. 42, of the Medical and Physical Journal, I had the satisfaction to find, that the observations and cautions contained therein, not only answered the purpose of putting my brethren of the pharmaceutical profession on their guard against the swarms of informers, who if no notice had been taken of the probable consequences of the act in its then state, would have dealt destruction very extensively around, but have also had their share in being the means of bringing about a very important and salutary reformation of the act itself.

Not many days after that letter had made its appearance in public, I was waited on by a gentleman from the Stamp Office, who informed me that I had it in my power to render an essential service to those who were about to be employed in a revisal of the schedule of the Medicine Act, by giving my assistance in pointing out such articles as had been improperly introduced into it.

I attended this gentleman at his chambers in the Temple, at an appointed hour; and we had a long conversation on such clauses of the act as bore most heavily on the medical practitioner; at the conclusion of our conference, he put a schedule into my hands, requesting me to take it home, and strike my pen through the names of those articles which I was of opinion ought not to have been inserted.

In complying with this request, I did not content myself with merely striking my pen through the names of the exceptionable articles, but subjoined my reason to each, why I thought it ought not to stand; confining myself, (with the exception of two or three things) to the articles of the Pharmacopœia, and not meddling with many others which appertained to the province of the perfumer and confectioner.

The alarm excited by the provisions of the act, and the insertion in the Schedule of such names as *Spanish juice, refined liquorice, arrow root, Turkey rhubarb*, and many other things equally undeserving a place therein, soon arrested the attention of the druggists of London; meetings were held, committees were formed, and, as the first step, a Memorial to the Lords of the Treasury was drawn up, praying a suspension of such prosecutions as might be brought under this new act, for the dispensing of medicines not properly coming within the meaning of the act, but which were included in the schedule.

Various communications were in the mean time received by the Committee from different parts of the kingdom, on the uncertain state into which the dealers in medicine were thrown by the operation of the Medicine Act; and the the London Committee were looked up to by the associations formed in different parts of the kingdom, as the medium through which an amelioration of the act, and of their situation in consequence of the act, was to be hoped for.

The Memorial was laid before Mr. Vansittart, who most readily and obligingly appointed a day at the Treasury for an interview; and at the appointed time, a deputation of four members of the Committee waited on him, and had a conference with him of above two hours, during which time various points in dispute were fully entered into.

The hardships to which all who dealt in any of the articles set forth in the schedule, were exposed to from the inroads and perjuries of common informers were particularly dwelt on. Feeling the force of this observation, Mr. V. replied, that it should be proposed to Parliament that in future all informations under the Medicine Act should be prosecuted by the Commissioners of the Stamp Office, who would not encourage any vexatious suit, or endeavour to strain the act beyond its just or fair limits.

We represented to him the great disproportion of the penalty to the offence, and instanced the possibility of a shopkeeper being prosecuted to conviction in the sum of ONE HUNDRED POUNDS, *besides costs of suit,* for the unintentional fraud against the revenue of *one shilling,** and at the same time adverted to those articles in the schedule which could not by any means be construed into nostrums, or any way proper to be included in the schedule.

It was also urged, that the allowing six months to the informer to bring his action, was giving him too great a latitude for committing his depredations; as total ruin might ensue to a person who might unintentionally and unknowingly continue the sale of a stampable article, contrary to the statute, in all that time; and that we conceived, if the law should enact that the prosecution should be brought within *one* month, the ends of justice would be fully answered. To these, and other propositions, Mr. Vansittart attended with much attention and patience, but gave no specific answer; only took notes of them; and promised that a bill for amending the act should be brought

* Common Spanish juice, as may be remembered, was among the articles of the late schedule. A retail apothecary, or grocer, selling eight separate pennyworths of Spanish juice therefore, without affixing a three-halfpenny stamp on each, was liable to a penalty of *ten pounds* for each parcel; and if unlicenced, *twenty pounds more*, the penalty for selling without a Medicine LICENCE!

brought into the House of Commons with a new schedule, free of the objectionable articles, which should be printed, and sent to the Committee; and time allowed for consideration.

It certainly was never the intention of the Legislature to harrass or throw any difficulties in the way of the regular medical practitioner; and this both Mr. Vansittart, and the Commissioners of Stamps assured us; and it now appears evident, that such a schedule would never have passed the house, but in the "MOB* *of bills that pressed on the consideration of the last parliament, and when it was in its last agonies.*" The Commissioners indeed, did every thing that men could do; and issued instructions to those who are employed by the Stamp Office to enforce the observation of the laws, not to lay informations or prosecute on account of any articles in the schedule, which were now found to be improperly placed therein.

Pending these transactions, I had enough to do to read all the letters I received from every quarter, some much to the purpose, and others very silly, particularly three or four anonymous ones, which I believe came from some of that description whose harvest was spoiled by the timely developement of impending mischief; and I am the more inclined to be of this opinion, from the publication, just about the time, of a most insidious advertisement, which appeared in all the newspapers, stating that, "Regular apothecaries, chemists and druggists had nothing to dread, the legislature having paid particular attention to their situation, and made a clear distinction between them and the dealers in quack medicines."

To this curious assertion was subjoined the fourth and fifth sections of the act; and by this advertisement, which many looked on as genuine authority from the Stamp Office, numbers of people were lulled into a security, and sold without scruple, stampable articles without stamps or licence. The fallacy of this publication I took on me to expose, at a general meeting of the Chemists, &c. &c. held at the Crown and Anchor, October 12, shewing that it had neither head nor tail; the advertisement was not dated from the Stamp Office, nor authenticated by the signature of Mr. Beresford, Secretary to the Commissioners, as it would have been, if genuine.

But, an event soon happened, which shewed, that the fears I had expressed, and the cautions I had given, were not

* Sir William Scott's Speech, April 6, 1803.

not in vain; and that the anonymous authors of private letters and of public advertisements, tending to mislead, were equally mistaken.

Although the Commissioners of Stamps, much to their honour, did their best to prevent the prosecution of individuals for the sale of what I term *innocent* articles, this did not prevent OPPOSITION COACHES from being started; and those informers, not connected with the Stamp Office, saw no reason why they might not run their machines, for a short time at least.

On a certain day, notice was given to some of the members of the Committee, that several informations under the New Medicine Act were to be tried before the Lord Mayor (Sir John Eamer), and that it was reported, in case of convictions upon these informations, the penalties would not amount to less than from four to five hundred pounds.

None of these were laid by any of the people connected with the Stamp Office; this was a "separate concern."

Some four or five members of the Committee made it their business to attend at the appointed time. The period being arrived, in waddled a fat, chubby figure, the informer's witness, bending under a huge wallet as large as the bag of a Jew old cloaths man, stuffed with various articles purchased from unwary dealers, each article carefully marked and labelled with the name of the offender, date, place, and every other circumstance; and from the bulk of the wallet, we were inclined to think, report for once might speak truth to the number of informations to be laid.

The first article produced was one ounce of refined liquorice, wrapped in brown paper, sold by a grocer in Houndsditch.

The majority of those who were summoned to appear, were grocers and confectioners, there were also one or two WIDOWS of apothecaries, who, to preserve life and soul, struggled to keep together the business of their deceased husbands until a purchaser could be found. There were no apothecaries nor druggists summoned; for informers always attack the most vulnerable, that is, those who are most likely to be ignorant of the law, and least likely to contend with them.

Where so productive a harvest was expected, in case of conviction on such a multitudinous budget of qui tam informations, the informer could easily afford the expence of employing counsel. An eminent barrister urged the suit, on behalf of the plaintiff. On the part of the defendant, Mr. Estcourt, the Solicitor of the Stamp Office, attended. We now saw, for the first time, an officer of the crown in a new a new and perhaps unprecedented situation; not standing by to see that the law was enforced upon the offenders against a revenue act, but as the advocate for those who had infringed it. His pleadings however were not directed to overturn the arguments of the learned counsel; these were unanswerable; but rather, to act as a mediator, and take off the blame from the defendant, whom he represented as having acted under the sanction of the Commissioners of the Stamps, who had informed a person deputed by the confectioners, that no prosecutions would be brought against persons selling such and such articles, not being medicines, although specified in the schedule. The learned counsel contended, and justly, that it was no matter to him in respect of his client, whether the law was a good one, or founded in error, it was sufficient such a law existed, and no legal power, not even the Lords of the Treasury themselves, could set aside an act of parliament.

I cannot see on what ground the Lord Mayor could possibly have avoided convicting the defendant, but for an accidental circumstance. One of the informations lay on the table, which Mr. Complin of Bishopsgate Street, who sat next to me, was reading, while I looked over his shoulder; I observed, that in one part of it, certain very material words, essential to constitute the validity of the information, had been omitted.

Whatever certainty is required in an indictment, the same is necessary in a qui tam information;* and consequently, as all the material parts of the crime must be precisely found in one, so must they precisely be *alleged* in the other. The information only stated that A. B. had sold one ounce of refined liquorice, contrary to the act; but, the material allegation, or averment, namely, its "*being a medicine used for the prevention, cure, or relief of a certain disease, ailment, complaint, &c. &c. incidental to the human body,*" was totally omitted. I communicated this discovery to Mr. Newman,† who agreed with me that it was fatal; and immediately shewed the Lord Mayor that the business was at an end; the information was of course quashed; and as all these summonses and informations were alike deficient in not having those material words of the act, every one of the other informations fell to the ground.

[ To be continued. ]

---

* Burn. † Clerk to the Lord Mayor.

